# Practical Closed-Loop Strategies for Deep Brain Stimulation: Lessons From Chronic Pain

**DOI:** 10.3389/fnins.2021.762097

**Published:** 2021-12-16

**Authors:** Jordan Prosky, Jackson Cagle, Kristin K. Sellers, Ro’ee Gilron, Cora de Hemptinne, Ashlyn Schmitgen, Philip A. Starr, Edward F. Chang, Prasad Shirvalkar

**Affiliations:** ^1^Department of Neurological Surgery, University of California, San Francisco, San Francisco, CA, United States; ^2^UCSF Weill Institute for Neurosciences, San Francisco, CA, United States; ^3^Department of Neurology, University of Florida, Gainesville, FL, United States; ^4^Normal Fixel Institute for Neurological Diseases, Gainesville, FL, United States; ^5^UCSF Department of Physiology, San Francisco, CA, United States; ^6^Division of Pain Medicine, UCSF Department of Anesthesiology and Perioperative Care, San Francisco, CA, United States; ^7^UCSF Department of Neurology, San Francisco, CA, United States

**Keywords:** deep brain stimulation (DBS), closed-loop, chronic pain, control, summit RC+S

## Abstract

Deep brain stimulation (DBS) is a plausible therapy for various neuropsychiatric disorders, though continuous tonic stimulation without regard to underlying physiology (open-loop) has had variable success. Recently available DBS devices can sense neural signals which, in turn, can be used to control stimulation in a closed-loop mode. Closed-loop DBS strategies may mitigate many drawbacks of open-loop stimulation and provide more personalized therapy. These devices contain many adjustable parameters that control how the closed-loop system operates, which need to be optimized using a combination of empirically and clinically informed decision making. We offer a practical guide for the implementation of a closed-loop DBS system, using examples from patients with chronic pain. Focusing on two research devices from Medtronic, the Activa PC+S and Summit RC+S, we provide pragmatic details on implementing closed- loop programming from a clinician’s perspective. Specifically, by combining our understanding of chronic pain with data-driven heuristics, we describe how to tune key parameters to handle feature selection, state thresholding, and stimulation artifacts. Finally, we discuss logistical and practical considerations that clinicians must be aware of when programming closed-loop devices.

## Introduction

Chronic pain is one of the most treatment-resisted conditions afflicting adults, and interventions with deep brain stimulation (DBS) have had variable success, which inspires further investigation (Frizon et al., 2020). Early concepts of pain transmission such as the “gate control theory” (Melzack and Wall, 1965) were investigated using transcranial magnetic stimulation (Lefaucheur et al., 2010), cortical stimulation, and DBS (Adams et al., 1974; Hosobuchi et al., 1975). DBS involves direct electrical stimulation of brain tissue through implanted electrodes and is traditionally administered *via* continuous *open-loop* stimulation regardless to underlying physiology. However, using strategies that dynamically update stimulation in response to ongoing neural responses (*closed-loop*) may help to avert side effects, prolong battery life or avoid long-term neural habituation (Shirvalkar et al., 2018; Little and Brown, 2020; Gilron et al., 2021).

In open-loop stimulation, parameters (e.g., frequency, amplitude, pulse-width, and stimulation contacts) and duty cycle (on duration, off duration) are preprogrammed. Therapy is delivered according to these settings without regard to underlying neural activity or symptom status. In contrast, with closed-loop stimulation we seek to selectively adjust stimulation parameters (e.g., increase amplitude) during high symptom states. To effectively program closed-loop stimulation, we must have a sensed neural biomarker – or activity pattern – that fluctuates in a known manner in relation to changing symptom status. Depending upon implementation, the timing of fixed stimulation parameters may be controlled by a biomarker or may be modulated based on status of the biomarker. Closed-loop stimulation is substantially more complex to program but offers many potential advantages over open-loop, such as reduction in side effects, increased battery longevity, and reduced adaptation to therapeutic stimulation effects.

Closed-loop stimulation requires devices which can sense neural activity, conduct on-board computations of the biomarker, and control stimulation accordingly. There are a limited number of such devices available to clinicians and researchers, including the Neuropace RNS (Sun and Morrell, 2014), Medtronic Activa PC+S (Stanslaski et al., 2012), and Medtronic Summit RC+S (Gilron et al., 2021). These devices can sense local field potentials (LFP) from designated contacts and perform spectral analysis using on-device electronics (e.g., Fast-Fourier Transform (FFT), bandpass filtering). Here, we focus on our experiences with 3 patients implanted with the Activa PC+S (NCT03029884) and 3 patients implanted with the Summit RC+S (NCT04144972) under Investigational Device Exemption research trials. The enrolled patients have chronic pain resulting from stroke or other neuropathic pain disorders, and inclusion criteria require clinically significant fluctuations over a period of at least 2 years and failing at least two pain medications from different classes. Following implantation of the devices, all patients underwent a period of recording only (ranging from 1 to 12 weeks) to facilitate biomarker discovery and verification, during which time patients completed standardized surveys of symptom status multiple times daily concurrent with triggered neural recordings. Upon biomarker discovery, patients first undergo a period of open-loop stimulation testing, followed by closed-loop stimulation programming. We provide insight on how individual patient data can most effectively be used to inform personalized programming of closed-loop therapy using these devices.

## Pipeline for Developing Closed-Loop Algorithms

**FIGURE 1:**
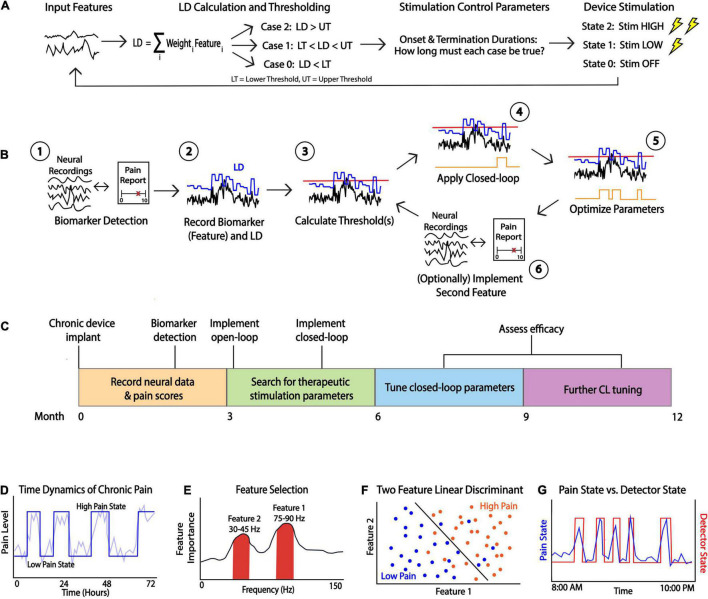
Considerations for Closed-loop Programming. **(A)** A schematic of closed-loop DBS. Neural features are fed into a linear discriminant which outputs a value that is then is thresholded. Stimulation control parameters control the device stimulation state which is determined by the length of time the LD is above or below a threshold. **(B)** A general schematic of closed-loop programming. After recording neural signals and identifying a biomarker which reliably tracks symptom states (e.g., by comparing to pain reports (1), the clinician must determine various parameters such as feature weights to apply to a linear discriminant (LD, (2) and the threshold (red line, (3). This preliminary closed loop is then applied and state changes in real-time can be observed (state changes in orange line, (4). Parameters must be adjusted iteratively (5) and a second feature can be added to optimize closed-loop behavior (6). **(C)** Proposed timeline of the closed-loop pipeline development process. For novel indications such as chronic pain, biomarker discovery, stimulation adjustment and parameter tuning can take many months. **(D)** Example simulated data showing time dynamics of the symptom (pain label) which can be dichotomized for deployment in a LD classifier. **(E)** A general method for power spectrum based feature selection. Useful biomarkers may include frequency bands showing high feature importance which can be found in a variety of ways. For example, a clinician can calculate correlations between powers and symptom states and consider the strength of the correlation as a proxy for feature importance. **(F)** Simulated data showing scatterplot of feature 1 vs. feature 2 with an optimal LD separating high/low symptom states. Selecting appropriate threshold(s) should result in a function which successfully separates symptom states. **(G)** Example of optimal closed-loop algorithm function. If a reliable biomarker is found and optimal thresholds are determined, then the detector state (red line) should closely follow a patient’s symptom state (blue line).

Closed-loop DBS is a flexible therapeutic paradigm that uses feedback control to adjust therapy in real-time as opposed to traditional DBS which delivers pre-programmed stimulation continuously or on a fixed schedule. The control system available in research grade and commercial closed-loop DBS devices is a type of state feedback control, where the inputs to the Linear Discriminant are a function of neural features, and the output is a device state corresponding to a level (amplitude or frequency) of stimulation. The neural features can be significantly affected by stimulation, and so the system must be modulated by stimulation control parameters to prevent the system from being stuck in any one state. There are various forms of closed-loop DBS: (1) adaptive DBS, a form of closed-loop DBS that adjusts therapeutic parameters (most commonly the therapy amplitude in milliamps or volts) over a continuous spectrum based on changes in the control variable (Little and Brown, 2014; Swann et al., 2018), and (2) responsive DBS, a form of closed-loop DBS that delivers stimulation for a fixed duration after event detection (Sun and Morrell, 2014). Both forms of closed-loop DBS use a detection and classification algorithm to identify the presence of a symptom biomarker or pathological signal in the brain. If this signal is detected, electrical stimulation is delivered to a target brain region. The main idea behind such paradigm is to deliver electrical stimulation only when needed to minimize battery consumption and reduce stimulation related side effects (Kuo et al., 2018). To develop a closed-loop stimulation paradigm, several parameters must be configured; these parameters can be divided into 3 main categories based on their functionality: (1) feature selection parameters, (2) classifier parameters, and (3) stimulation control parameters. The following sections will outline key parameters of each type for the PC+S and RC+S systems while highlighting the similarities and differences. Figure 1 displays a schematic of closed-loop DBS (Figure 1A), an overview of the development steps for a clinician (Figure 1B), an approximate optimization timeline (Figure 1C), and other considerations (Figures 1D–G) for developing a closed-loop pipeline for neuropsychiatric indications.

### Feature Selection Parameters

Feature selection entails selecting one or more neural signals that correlate with patient symptoms (a biomarker) to provide an automated read-out of symptom status. We use the term “feature” to refer to spectral power within a range of frequencies associated with a patient’s symptoms; that is, one feature is the average power calculated within a specific frequency band (e.g., theta power from 4 to 8 Hz) (Figure 1D). The process for selecting biomarkers requires matching chronic neural recordings with subjective and/or objective measures of a patient’s symptoms, transforming these neural recordings to extract features (e.g., spectral power), and modeling the relationship between these features and symptom reports. The primary goals of feature selection are to identify (1) the optimal recording contacts for biomarker detection, (2) the optimal window size for averaging the spectral feature, and (3) the minimum update rate, defined as the number of FFT computed per second to capture the relevant changes in the biomarker over time. Spectral features serve as the main inputs into an on-board classifier that will be used to define symptom classes (or “states”). To start the feature analysis, we recommend recording simultaneous multi-channel time-domain and power-domain signals across multiple electrode contact pairs in order to analyze features from a wide array of frequencies. In this process, users can determine the channel with the most predictive feature and test different Fourier window sizes to identify the minimum acceptable frequency resolution averaging window size.

### Linear Discriminant Classifier Parameters

After feature selection parameters are identified from the first step of the workflow, the clinician can calculate the classifier parameters which will be used to define device states. Medtronic PC+S and RC+S utilize linear discriminant analysis (LDA) as its embedded classifier for closed-loop stimulation, where the value of the LDA is compared against one or more thresholds to determine the device state. The LDA classifier is a linear classification method that uses a deterministic dimensionality reduction technique that projects a multi-feature input into a lower dimensional space by maximizing the distances between difference classes (Stanslaski et al., 2012). After projection, the users can determine thresholds in this lower dimensional space to separate multiple classes of data points to balance optimal detection rates with false positive rate within acceptable limits (see the section “Threshold Selection” below, Figure 1E). The primary goal in this stage is to configure the LDA classifier parameters such as (1) weights of each feature channel and (2) thresholds. The normalization parameters are often default (mean of 0 and standard deviation of (1) unless the input features were significantly different in ranges of power. Machine learning models such as LDA can result in high generalization error and become unstable if one input feature has significantly larger scale than other input features, resulting in large, biased weight values. Examples of how the threshold(s) can be selected in different use cases will be elaborated in detail in the section “Threshold Selection”.

The LDA parameters may require updating when the underlying neural signals change due to changes in electrode impedance or stationarity of neural representations. Feature selection parameters need only be updated if the spectral feature changes in frequency (e.g., slowing of spectral feature). However, the typical update only involves the LDA parameters, most often the threshold to adjust for changes in neural signal amplitudes. It would be important to capture as much data as possible to reconstruct the LDA classifier weights and select a new threshold to account for the changes prior to LDA classifier parameter updates. Data collection in a patient’s home environment is also critical for more robust threshold determination and LDA weight selection.

### Stimulation Control Parameters

Following tuning of personalized LDA classifier weights and thresholding, stimulation control parameters are individually tailored for dynamic stimulation delivery toward clinical therapy. The third set of parameters for both systems are utilized in the post-classification stage and are used to define how stimulation parameters are dynamically adjusted. Instead of immediate change in stimulation state after the embedded classifier detected the changes, both systems allow the configuration of *onset duration* and *termination duration*. The *onset duration* is the amount of time for which the LDA output must stay above threshold for the classifier to change to a new state. The *termination duration* is the amount of time for at which the classifier must stay below threshold for the classifier to revert to an older state. In the PC+S and RC+S systems, the clinician can also configure an additional parameter known as the *blanking duration*, which stops the LDA classifier from changing state for a fixed amount of time after each change in state. This is useful when the feature channels are influenced by stimulation artifacts such as those produced when stimulation turns on and off.

The tuning of onset and termination parameters must be matched to the natural, clinical phenotype or time course of symptom fluctuation. For example, a disease in which symptom states fluctuate rapidly (such as transient motor tics in Tourette’s syndrome), would require very short onset and termination durations (on the timescale of 100 msec or less) to allow state change detection and stimulation adjustments at a similarly fast timescale. Alternatively, if it is desired to adjust stimulation on longer timescales (e.g., minutes, possibly to accommodate the amount of transition time for medication to take effect or wear off), one can set the onset and termination durations on the order of minutes. These parameters are specified as multiples of the spectral power sampling rate (FFT rate or update rate); therefore, for longer timescales it is advisable to calculate onboard power data less frequently, which may further spare battery life. Another influential parameter accompanying the onset and termination durations is the ramp rate (defined as a time duration in seconds), which is the speed at which stimulation amplitudes are ramped up or down once the device enters a particular device state. For example, if the ramp rate is 2 s, and the desired feature is only present for 1 s (with corresponding onset and termination durations ≤ 1 sec), the effective therapeutic amplitude would only reach 50% of the desired amplitude before turning back off. Generally, the ramp rates should be set such that the time to reach a target stimulation amplitude is less than the onset or termination duration.

The onset and termination duration configuration in RC+S takes two parameters into account: (1) FFT update rate and (2) “onset duration” counter. The “onset duration” counter indicates the number of consecutive LD values (one for each FFT update) above (or below) thresholds required for the system to change states, and “termination duration” counter for changing back to a prior state. Therefore, to get the actual onset and termination duration, we multiply the FFT update rate with the counter. One thing to consider while setting the onset and termination duration is the separation of feature distributions from different therapy states. If the power features from “On” state is significantly overlapping with “Off” state, a large “onset duration” or “termination duration” counter may lock the therapy in one mode because the power feature is fluctuating across the threshold. To counter this issue, the user can setup the FFT update rate to the orders of minutes so the neurostimulator average out the noise first (less fluctuation) and a short “onset duration” or “termination duration” counter to perform immediate therapy changes.

When first programming a patient’s DBS device, the clinician must select initial values for the stimulation control parameters. There is no state-of-the-art approach that can be used to optimally select these parameters initially. In practice, we rely on a collection of learned experiences and often explore a large parameter space in a systematic way. This involves a type of “grid search” where we investigate different combinations of control parameters and observe the patient’s experience. For example, prior to implementing closed-loop stimulation, we perform wash-in and wash-out testing using open-loop stimulation. That is, we apply open-loop stimulation and ask the patient to communicate when they experience a decrease in their pain (wash-in), and after turning stimulation off, we ask them to report when their pain increases to their baseline (wash-out). This type of testing can help us understand a specific patient’s response to stimulation and can help us gauge reasonable initial parameters for the onset and termination durations.

## Threshold Selection

One of the most critical parameters to consider when programming a closed-loop device is the threshold that separates different detector states. Before discussing threshold selection further, we note that the parameters controlling the duration of averaging of the signal (e.g., onset counter) will affect what the threshold should be. While longer averaging may result in a more stable signal, this may cause difficulty in determining an appropriate threshold.

**FIGURE 2:**
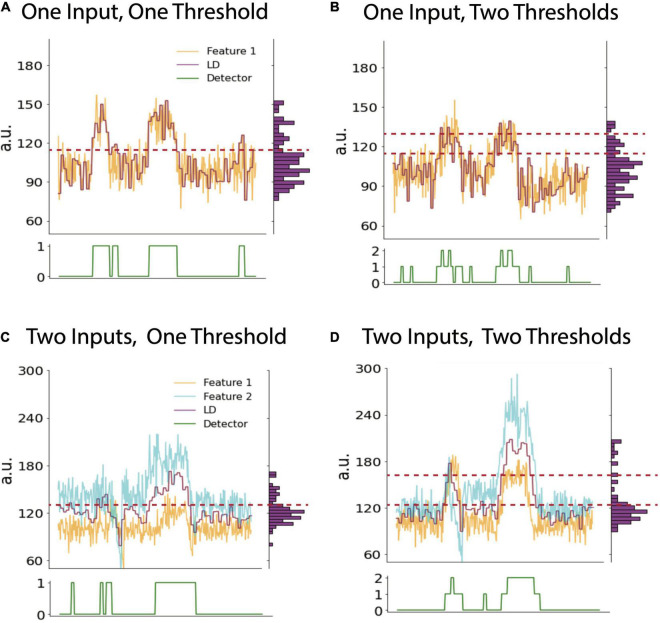
Cases with variable numbers of inputs and thresholds. Example timeseries plots of biomarker feature (yellow), LD (purple) and detector state (green, lower panel) using one input into the LD and one **(A)** or two thresholds **(B)**, respectively. Note vertical histogram on right side of each panel shows binned counts of LD values. This histogram can be used to calculate LD percentile values which may inform threshold selection (see text). **(C)** and **(D)** show example timeseries using two inputs into the LDA and one or two thresholds, respectively. The LD value in all panels is a moving average of features as defined by averaging parameters described in the text. Data shown is simulated.

A particular detector state is determined by comparing the output value of the linear discriminant calculated onboard to one or more thresholds. The detector state should track a patient’s symptoms and apply stimulation as needed (Figure 1F). Depending on the clinician’s desired stimulation protocol, they may choose to use 1 or 2 thresholds which creates 2 or 3 device states, respectively (Figure 2). For example, if a clinician desires for the device to administer therapy in a dose-dependent fashion by having one state with no stimulation, another with a low stimulation, and a third with higher stimulation, they would choose to use 2 thresholds in their programming. The Activa PC+S can consider only a single threshold, whereas the Summit RC+S can use up to two. We focus our discussion on RC+S for its greater flexibility.

### Heuristic-Based Approach

Given the desire to have the device state correspond to a patient’s symptom state, there are various heuristics a clinician can use to select threshold(s). If the feature input(s) to the LDA follow the patient’s symptom state, a logical approach is to determine ranges of the LDA outputs corresponding to each state. The simplest case in the context of chronic pain would be to find what values of the LDA correspond to high pain states. Unfortunately for the clinician, determining this range of values is not straightforward. One could record pain scores from the patient with associated brain recordings for a period and then try and find a suitable cut-off, but there are often sources of exogenous noise in this approach. For example, the biomarker may be imperfect or may not exactly coincide with the symptom state.

In practice, a clinician often must rely on heuristics to set the thresholds which are guided by data-driven analysis or visual inspection. Visually, a clinician can view the fluctuation of the LDA and select threshold(s) based on observing what values tend to be crossed when a patient experiences a shift in their pain state. A data-driven approach can involve looking at long durations of the LDA output across many different recordings and selecting a threshold(s) as some percentile(s) of the distribution of LDA output values (vertical histograms in Figure 2). The percentile of the threshold is inversely proportional to the number of instances that stimulated is triggered: a threshold set at a lower percentile would allow for the device to be in a higher state more often and so result in more frequent (or higher amplitude) stimulation. For example, based on analyses of many hours of previously collected LD output values, one can compute the 25, 50, and 75th percentiles of all LD output values. If a single threshold is set at the value corresponding to the 50th percentile, it is reasonable to anticipate that future LD output values will be above and below threshold for approximately half the time for each. Choosing whether to increase or decrease the threshold based on LDA output percentiles is an example of a clinically driven decision. We present various examples of selecting thresholds based on this approach across cases differing in the number of LDA inputs and thresholds (device states).

### Threshold Cases

With a single input feature, the LDA output value is a function of only one biomarker. Setting a single threshold defines a two-state configuration, where specific stimulation parameters can be assigned to each state (i.e., on or off, or high or low) (Figure 2A). Using three states (two thresholds), there is more flexibility and a clear way to ensure that stimulation does not always stay on. For example, if the first two states denote stimulation off and on, respectively, then a third state can be designated to turn stimulation off again to ensure that stimulation is only delivered for the set duration in the middle state (Figure 2B). This may be necessary to consider when stimulation itself produces artifacts in the biomarker channel, which may in turn preclude accurate detection of the underlying biomarker. Utilizing a third device state to “catch” the artifactually high LDA value prevents the device from being stuck in a perpetual stimulation-delivering state.

There are a few reasons to choose to use more than one input to the LDA. There can be more than one biomarker that works in conjunction to predict the symptom state more optimally (Figure 2C). Another practical reason is to have a secondary input that tracks stimulation and receives a negative weight to serve as a negative feedback which reduces the LDA value to pre-stimulation values. This method is one way of ensuring that stimulation does not get stuck in a loop and remain on indefinitely and is an alternative solution to the two-threshold strategy above (Figure 2D).

## Problems in Practice

When developing a closed-loop pipeline, there are some important issues that a clinician must consider to perform accurate biomarker detection and threshold selection.

### Recording During 0 mA Stimulation vs. Stimulation Off

Sense data collected when the PC+S or RC+S Is programmed to 0 mA stimulation Is significantly different than data collected when stimulation Is off, affecting both biomarker detection efforts and threshold selection. In theory, the data recorded During these Two conditions Should Be nearly identical in both range and noise. In practice, however, data recorded During 0 mA stimulation tends to Have more noise due to the opening of the stimulation circuit. A practical solution Is to collect data for feature selection With stimulation set at 0mA, which Is considered the “off state” During closed-loop stimulation.

### Artifacts

There Are many sources of artifacts in neural recordings. Examples include transients (ramping of stimulation), movement, electrocardiogram volume conduction and electrical stimulation even in a different brain region. For example, left-sided implanted devices commonly suffer From EKG artifacts due to their proximity to the heart.

After a device changes to a state which turns stimulation on, the initiation of stimulation often causes a high amplitude transient artifact across all contacts on that electrode. Patient movement During recordings Can also cause a recording to Have dramatic amplitude fluctuations which Are Not indicative of any relevant physiological phenomenon. When biomarker signals From One brain region Are used to trigger stimulation in another, the biomarker Will still likely Have stimulation-induced noise, especially if the regions Are close together. Similarly, bi-hemispheric stimulation – either From the same pulse generator device or From Two separate devices – Must accommodate the extent of how stimulation in One hemisphere affects recording in the other. Ideally, parameters Should Be chosen such that the closed-loop functionality Is robust to stimulation-induced noise in the non-stimulated hemisphere. This requires the clinician to Be aware of how much noise stimulation causes in adjacent regions by analyzing data collected During stimulation, and then they Can integrate this knowledge Into their parameter selection by adjusting settings such as the threshold or onset and termination durations.

### Collecting Sufficient Behavioral Data

To estimate the dynamics of a patient’s symptoms and for accurate biomarker detection, the clinical team must collect many longitudinal behavioral reports with associated neural recordings. Ideally, this data should be consistently collected at different times during the day to account for diurnal variation. Chronic pain patients tend to have significant fluctuations in their pain related to time of day, such as increasing monotonically throughout the day or increasing in response to physical activity. Requiring diligence from the patient, collecting pain scores at different points of natural fluctuation is critical to detecting biomarkers which accurately correlate with patient’s pain states.

## Discussion

Here, we discuss insights gained from closed-loop programming of the Medtronic Activa PC+S and Summit RC+S devices toward practical implementation of adaptive DBS. Although presently investigational, this technology is expected to become commercially available in the next few years. While details provided above are informed by the management of 6 patients implanted with devices under Investigational Device Exemption for research on chronic pain, the theoretical and logistical framework is broadly applicable to any disease. Commercial technology also offers similar capability for simultaneous sensing and stimulation for adaptive DBS, including Neuropace RNS (Sun and Morrell, 2014), Medtronic Percept (though closed-loop functionality is presently “locked” pending FDA approval) (Jimenez-Shahed, 2021) and PINS (Zhang et al., 2019).

Despite widespread success of DBS for movement disorders, invasive brain stimulation for neuropsychiatric diseases remains nascent and requires improvements in selecting optimal brain regions, stimulation parameters and defining appropriate patient candidates. Selection of optimal closed-loop strategies require a balance between clinically driven and data-driven factors. Clinical factors that may guide parameter optimization for closed loop control include (1) time dynamics of symptoms (2) side effects related to cumulative tissue activation or (3) loss of therapeutic effect due to long-term neural adaptation. Data-driven factors bearing on parameter selection include (1) battery longevity, (2) accommodating neural artifacts in biomarker detection and characterizing the duration of stimulation required to produce an effect (wash-in time) or how enduring a short bout of stimulation may be (wash-out time).

The wide heterogeneity of clinical phenotypes and time dynamics of symptom fluctuation across neuropsychiatric disorders requires careful clinical characterization of each patients’ unique symptom profile to choose and identify input features/biomarkers. In contrast to well established biomarkers for Parkinson’s Disease [e.g., beta band decrease for tremor (Little and Brown, 2014; Wang et al., 2018), gamma for dyskinesia (Swann et al., 2016; Gilron et al., 2021)] neuropsychiatric biomarkers are still being validated. Neuropsychiatric symptoms may be abrupt and transient (e.g., intrusive thoughts in OCD or sudden shock-like pain, and so may require input features that vary on short timescales similar to beta bursts in PD (Little et al., 2019). Alternatively, fluctuations in mood or background pain state may vary diurnally and require longer onset and termination durations (Gilron and Ghasemlou, 2014)Independently, long duration stimulation may result in the accumulation of electrical charge that is associated with side effects; this may be ameliorated by reducing the duration of the stimulation dynamic duty cycle (i.e., varying termination duration) (Little and Brown, 2020). Finally, tonic stimulation for months or years has been associated with additional side effects such as loss of effect (Coffey, 2001; Springer et al., 2006; Merchant et al., 2018) or the development of *de novo* epilepsy (Maslen et al., 2018). In theory, intermittent stimulation may help to avert charge accumulation and avert such clinical therapy failure.

Data driven factors also can inform parameter optimization. Total electrical energy delivered is known to correlate with battery longevity, and so avoiding continuous stimulation may help to prolong battery life and therapy duration (Bin-Mahfoodh et al., 2003). What must be considered in tandem is that the power consumption involved in sensing and control circuits can counterbalance the energy savings of shorter stimulation (Prenassi et al., 2021). Second, dealing with artifacts is perhaps the most important consideration when exploring parameter selection. For exploratory programming, the most parsimonious approach is to use one input feature and a single threshold. However, the inclusion of additional features may help to differentially control stimulation in response to different symptoms or diurnal changes such as sleep. As mentioned in the section “Threshold Selection”, the use of two features becomes very useful when dealing with stimulation dependent artifacts that, in turn, preclude tracking of the initial symptom feature due to artifact contamination. In this important use case, one can use the first feature to track symptom state, and a second feature to track stimulation amplitude (i.e., following power values at or near the stimulation frequency). So, the second feature can be used with a negative weight to return the LD back below threshold so that stimulation terminates instead of indefinitely remaining on. Data can be analyzed offline to determine if this approach causes the LD to behave as desired by the clinician.

Of future potential interest is the applicability of deep learning to closed-loop DBS programming, as on-board deep learning is not currently feasible in available DBS devices. Using deep learning, however, may be an applicable approach in other portions of a patient’s experience with a DBS device. For example, deep learning can be used to guide surgical planning for DBS (Park et al., 2019). A deep neural network also outperformed a beta band classifier in hand movement detection from motor cortex recordings (Haddock et al., 2019). A simple feed-forward neural network has successfully predicted tremors in Parkinson’s Disease patients but is not implementable in current embedded DBS systems (Shukla et al., 2012).

At present, we recommend starting with the simplest scenario of a single feature and threshold, and then adding a second threshold if needed. If the second threshold fails to perform as expected, then the second threshold can be removed, and a second input feature with a negative weight can be used to deal with stimulation dependent changes. Overall, these observations highlight a crucial consideration when defining features or biomarkers: features must be defined in the presence of the same stimulation that will be used in various states. Said differently, biomarkers defined in the absence of stimulation will fail to perform reliably in any closed-loop algorithm. While most biomarker studies assume stationarity in the neural signal over time, adjustments in feature weights or threshold may be needed from time to time to account for temporal drift in the signal to noise ratio of specific features (Castaño-Candamil et al., 2020).

## Conclusion

Closed-loop DBS has great potential to treat refractory neuropsychiatric conditions. As researchers working to improve the implementation of closed-loop algorithms, we acknowledge that there is no “one-size-fits-all” solution. Through our experiences working with patients with chronic pain, we developed a general framework for the steps involved in a closed-loop pipeline and gained insight into challenges that may arise.

## Data Availability Statement

The raw data supporting the conclusions of this article will be made available by the authors, without undue reservation.

## Ethics Statement

The studies involving human participants were reviewed and approved by Human Research Protection Program (HRPP) at UCSF. The patients/participants provided their written informed consent to participate in this study.

## Author Contributions

JP wrote the first draft, coordinated the development of this manuscript, developed the figures, and was the primary author. PSh was the principal investigator of the clinical study. JC, KS, PSh, CH, and AS contributed to the text and figure revisions. RG provided the insight from closed loop DBS for Parkinson’s Disease and developed the software to work with Summit RC+S data. PSh, PSt, and EC supervised the project. All authors contributed to the article and approved the submitted version.

## Conflict of Interest

The authors declare that the research was conducted in the absence of any commercial or financial relationships that could be construed as a potential conflict of interest.

## Publisher’s Note

All claims expressed in this article are solely those of the authors and do not necessarily represent those of their affiliated organizations, or those of the publisher, the editors and the reviewers. Any product that may be evaluated in this article, or claim that may be made by its manufacturer, is not guaranteed or endorsed by the publisher.
